# Innovations for colonic endoscopic submucosal dissection: combination of the latest game changers

**DOI:** 10.1055/a-2224-8465

**Published:** 2024-02-28

**Authors:** Mathieu Pioche, Louis Jean Masgnaux, Romain Legros, Timothée Wallenhorst, Jérémie Albouys, Jérôme Rivory, Jeremie Jacques

**Affiliations:** 1 Gastroenterology and Endoscopy Unit, Edouard Herriot Hospital, Hospices Civils de Lyon, Lyon, France; 2 Gastroenterology and Endoscopy Unit, Dupuytren University Hospital, Limoges, France; 3 Gastroenterology and Endoscopy Unit, Pontchaillou Hospital, Rennes, France

Endoscopic submucosal dissection (ESD) in 2023 is nothing like it was originally in 2001, with major changes in equipment and strategies. However, the spread of ESD has been slow because it is a challenging procedure, and major new developments are expected to simplify it further.

We present a case of ESD of a nongranular laterally spreading tumor in the right colon angle, using a combination of the latest technological developments ( Fig. 1Fig. 1 , Video 1Video 1 ) to simplify the main difficulties of the procedure.

**Fig. 1:**
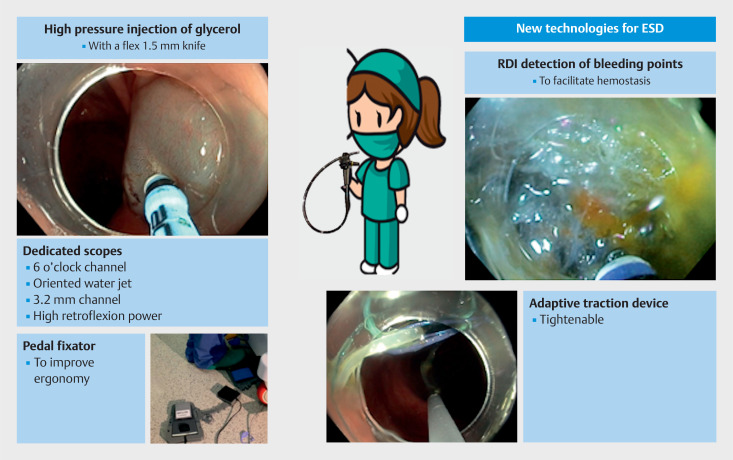
**Fig. 1** New game changers for endoscopic submucosal dissection. Flex knife for high pressure jet injection of macromolecules. Adaptive multipolar traction. Improved ergonomy with a pedal fixator. Red dichromic imaging (RDI) for the detection of bleeding points.

New game changers for endoscopic submucosal dissection.Video 1Video 1

We used a pediatric therapeutic colonoscope (PCF 190L; Olympus, Tokyo Japan) with a 3.2-mm operating channel located in the 6 o’clock position with waterjet function. While high-pressure injection of macromolecules was possible with some devices 11 such as the Erbe jet (Erbe, Tübingen, Germany) 22 , the rigidity and size of the knife made dissection of the very thin colonic (rectal) submucosa difficult. With the arrival of the new Hybrid flex knife (Erbe), the flexibility and finesse of the electrode are now close to those of the most commonly used colonic knives (e.g. DualKnife; Olympus), and allow application of high pressure to the colon. However, exposure remains a key factor, and the use of a multipolar adaptive traction device (ATRACT; Hospices civils de Lyon, Lyon, France) remains essential for creation of triangulation and adjustment of traction during the procedure 33 . Ergonomics is surely also one of the keys to this procedure, and pedal management needs to be improved to avoid pedal errors and to make it easier to locate the correct pedal without the need to look down at the feet. To this end, the IPEFIX pedal fixator has already demonstrated its effectiveness in reducing the number of pedal errors, and the need to look at the feet, in a prospective randomized study 44 .

Finally, managing bleeding through early detection is a key factor in reducing operating time and operator fatigue, and in this respect, red dichromic imaging (RDI; Olympus) is a valuable aid in achieving hemostasis quickly and reducing endoscopist stress 55 .

New game-changing innovations for ESD and their combined use could improve the accessibility and dissemination of the technique, without skipping organized and effective training.

Endoscopy_UCTN_Code_TTT_1AQ_2AD

Zitierweise für diesen Artikel

Endoscopy 2023; 55: E1172–E1173. doi: 10.1055/a-2191-5546

## Notes

Endoscopy E-Videos https://eref.thieme.de/e-videos
*E-Videos* is an open access online section of the journal *Endoscopy* , reporting on interesting cases and new techniques in gastroenterological endoscopy. All papers include a high-quality video and are published with a Creative Commons CC-BY license. Endoscopy E-Videos qualify for HINARI discounts and waivers and eligibility is automatically checked during the submission process. We grant 100% waivers to articles whose corresponding authors are based in Group A countries and 50% waivers to those who are based in Group B countries as classified by Research4Life (see: https://www.research4life.org/access/eligibility/ ). This section has its own submission website at https://mc.manuscriptcentral.com/e-videos .
